# Cognitive Impairment among Cardiac Arrest Survivors in the ICU: A Retrospective Study

**DOI:** 10.1155/2019/2578258

**Published:** 2019-11-03

**Authors:** Soo Hyun Kim, Sang Hoon Oh, Kyu Nam Park, Taek Hun Kim

**Affiliations:** ^1^Department of Emergency Medicine, Eunpyeong St. Mary's Hospital, College of Medicine, The Catholic University of Korea, Seoul 03312, Republic of Korea; ^2^Department of Emergency Medicine, Seoul St. Mary's Hospital, College of Medicine, The Catholic University of Korea, Seoul 06591, Republic of Korea

## Abstract

**Background:**

Recent studies have presented the effects of cardiac arrest on long-term cognitive function and quality of life. However, no study has evaluated cognitive function in the early stage after regaining consciousness.

**Purpose:**

The objectives of this study were to analyse the incidence, clinical course, and associated factors of cognitive impairment of cardiac arrest survivors in intensive care unit (ICU).

**Patients and methods:**

We administered the Mini-Mental State Examination (MMSE) to cardiac arrest survivors who were treated with targeted temperature management (TTM) immediately after regaining consciousness. Patients whose MMSE scores indicated impaired cognitive function (MMSE < 24) were retested before ICU discharge.

**Results:**

In 92 patients, the median MMSE score was 21.0 (interquartile range (IQR), 16.0–24.0), and cognitive impairment was found in 64 patients. Fifty-three patients completed follow-up MMSEs, and the median scores were 20.0 (IQR, 13.5–23.0) for the first and 25.0 (IQR, 21.5–28.0) for the last test. Of the specific domains, recall (0.0 (IQR, 0.0–1.0) to 2.0 (IQR, 1.0–3.0)) and attention/calculation (3.0 (IQR, 1.0–4.0) to 4.0 (IQR, 2.0–5.0)) were the most affected domains until ICU discharge. The factors that were correlated with cognitive impairment on the last MMSE were older age (OR, 1.07 (95% CI, 1.01–1.14), *p*=0.016), increased time to return of spontaneous circulation (ROSC) (OR, 1.08 (95% CI, 1.02–1.15), *p*=0.012), and length of hospital stay (OR, 1.07 (95% CI, 1.00–1.14), *p*=0.044).

**Conclusions:**

Cognitive impairments were common immediately after patients regained consciousness but recovered substantially before ICU discharge. Recall and attention/calculation still were impaired until ICU discharge, and older age, increased time to ROSC, and LOS were associated with this cognitive decline.

## 1. Introduction

Cardiac arrest is a major health problem [1] and has a yearly incidence of approximately 50–110 per 100000 people worldwide [2]. However, 30% to 50% of cardiac arrest survivors were reported to suffer from cognitive impairment [3]. Memory, attention, and executive functions were most affected [3, 4], followed by negative effects on participation/autonomy and on quality of life [5, 6]. Although targeted temperature management (TTM) has contributed to an overall increase in cardiac arrest survival and good neurological recovery over the past decade [7, 8], recent studies have presented that similar long-term cognitive impairments were found in cardiac arrest survivors treated with TTM [9, 10].

Regarding long-term cognitive impairments, there was a high heterogeneity for duration of follow-up between the studies [3, 9–11]. However, no study has evaluated cognitive function in the intensive care unit (ICU) early after regaining consciousness, and the process of cognitive function recovery has not been entirely presented. Further information on the quantification of recovery from cognitive impairment immediately after awakening would be important for counselling families in the ICU and useful as baseline for monitoring recovery progress in future interventional trials aimed at reducing cognitive impairment.

There are many of tools used to assess cognitive function after cardiac arrest but they are not yet standardized. Researchers use different tools as follows: one study used the tools of the 41 Cent Test, the Montreal Cognitive Assessment (MoCA), and the Computer Assessment of Mild Cognitive Impairment [12] and the other study used a neuropsychological test battery including Cognitive-Log, Trail Making Test A and B, Verbal Fluency Test, Paragraph Recall Test Direct and Delayed, and the Adult Memory and Information Processing Battery Task A [13]. And, some group used other tools as follows: the Rivermead Behavioural Memory Test (RBMT), the Frontal Assessment Battery, and the Symbol Digit Modalities Test [9].

Among them, the Mini-Mental State Examination (MMSE) is a standardized tool for bedside assessment of overall cognitive function and was originally developed to screen for dementia and delirium [14]. The MMSE consists of a questionnaire that covers six cognitive domains. The maximum score is 30, and the score differs greatly depending on the educational level or age of the subject, but generally, values below 24 indicate cognitive impairment [14]. Sometimes, the MMSE is used as a screening instrument for cognitive impairment in cardiac arrest survivors because it is simple, widely used, and practical for serial use, and the validity of the MMSE is well established [5, 15, 16]. We administered the MMSE to TTM-treated cardiac arrest survivors who regained consciousness. The patients who presented impaired cognitive function were retested later during ICU care.

The aim of this study was to analyse the incidence and clinical course of cognitive impairment in TTM-treated survivors who regained consciousness during intensive care. We also evaluated the associating factors for early cognitive impairment, especially the degree of neurological injury using serum biomarker in these patients.

## 2. Methods

We conducted a retrospective analysis of one tertiary hospital TTM registry and collected adult cardiac arrest patients (>18 years old) treated with TTM starting in 2009. During study period, a total of patients who regained consciousness after TTM between March 2009 and June 2017 were recruited. We excluded patients who did not undergo the MMSE during the admission period. The study was approved by our Institutional Review Board and was conducted with a waiver of patient consent because of the noninterventional, retrospective design of the study.

### 2.1. TTM and Sedatives Protocol

During the study period, postcardiac arrest care including TTM was performed according to the guidelines that were current at the time of treatment [17, 18]. Once a patient achieved return of spontaneous circulation (ROSC), the patient was considered eligible for TTM at 33°C, which was initiated as soon as possible after ROSC regardless of the initial cardiac rhythm or arrest location. In our TTM protocol, midazolam (0.08 mg kg^−1^ intravenously) was immediately administered during induction to control shivering followed by a continuous midazolam infusion (0.04–0.2 mg kg^−1^ h^−1^). After completion of the 24-h maintenance period, controlled rewarming at a rate of 0.25°C h^−1^ was performed until the core temperature reached 36.5°C. Midazolam dosage was reduced during rewarming and stopped before reaching normal body temperature.

### 2.2. MMSE Measures and Other Variables

According to our protocol, after patients who presented good neurological function were extubated, time at which a meaningful MMSE could be conducted; cognitive function was assessed by ICU physicians. Individuals with an endotracheal tube or who were essentially muted did not undergo an MMSE. In this study, the MMSE standardized in Korean was used [19]. The MMSE consists of a thirty-point scale (range 0–30; 30 = max) that assesses six different cognitive elements or domains: (1) orientation to time (range 0–5), (2) orientation to place (range 0–5), (3) three word registration (range 0–3), (4) attention/calculation counting backwards by seven (range 0–5), (5) delayed recall of the three words (range 0–3), and (6) language involving comprehension of a three-step command, naming, repetition, and sentence writing (range 0–8), and visuoconstruction involving the copy of intersecting pentagons (range 0–1). Whenever possible, MMSE in cognitively impaired patients (MMSE < 24) was reassessed by same physician on ICU discharge day.

Demographic information, resuscitation variables, and comorbidities such as coronary artery disease (CAD), hypertension, and diabetes mellitus (DM) were analysed from the patient registry. We also evaluated some confounding variables for MMSE score. The total dose of midazolam administered to patients during TTM was analysed. The time to MMSE was defined as the median number of hospital days on which the MMSE was conducted, and the time to obey was recorded when the patient gave a meaningful response to verbal commands.

During study period, neuron-specific enolase (NSE) and S100 calcium-binding protein B (S100B) were measured as standard tests. Initial measurements of NSE and S100B were obtained as soon as possible after ROSC, and these measurements were repeated 24, 48, and 72 h later based on time of ROSC. The serum was analysed with Roche Elecsys NSE and S100 reagents (Roche Diagnostics, Mannheim, Germany). If the serum showed significant haemolysis, the results for NSE were discarded. The upper limits of normal serum levels for NSE and S100B were determined by our laboratory as 14.7 ng mL^−1^ and 0.105 ng mL^−1^, respectively.

### 2.3. Statistical Analysis

All data are summarized and displayed as the mean ± standard deviation or median with interquartile range (IQR) for continuous variables and as the number (percentage) of patients in each group for categorical variables. Patients were separated by their MMSE scores from the first and last measurements into cognitively impaired (MMSE < 24) and cognitively intact (MMSE ≥ 24) groups. Comparisons of categorical variables between groups were made using either *χ*^2^ test or Fisher's exact test as appropriate. In addition, continuous variables were compared between groups using *t* tests or Mann–Whitney *U* tests. Associated factors were evaluated using multivariate logistic regression analyses, and odds ratios (ORs) with 95% confidence intervals (CIs) were estimated in the logistic regression models. For repeated measurements, the Wilcoxon signed-rank test was used. All analyses were performed using SPSS 24.0 software (IBM, SPSS Inc., Chicago, IL, USA). A value of *p* < 0.05 was considered significant for all analyses.

## 3. Results

**Figure 1:**
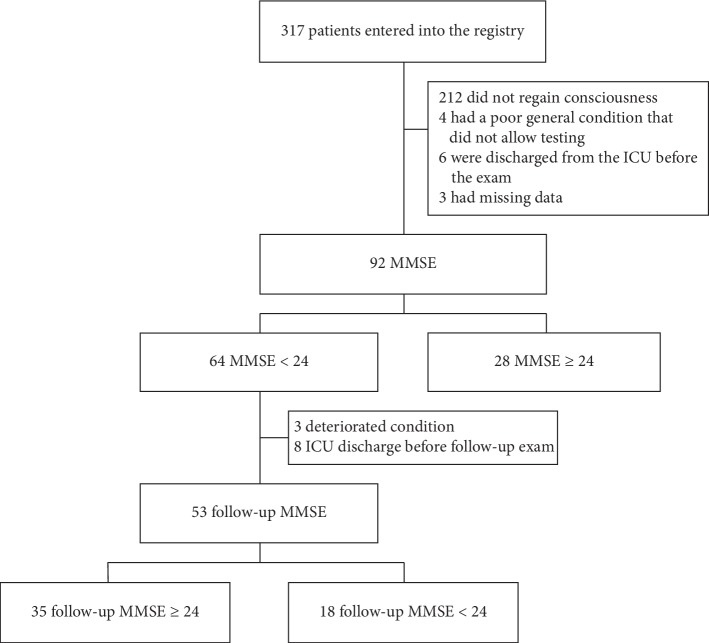
Flowchart of study inclusion.

During the study period, a total of 1280 adult patients were attempted cardiopulmonary resuscitation. Of these, 569 patients regained spontaneous circulation and 317 patients (55.7%) underwent TTM (Figure 1). Of these patients, 212 patients did not regain consciousness, and 13 patients were excluded because of ICU discharge before MMSE examination (*n* = 6), poor neurologic status (*n* = 4), or missing data (*n* = 3). Finally, 92 patients were included in this study. The baseline demographics, comorbidities, resuscitation variables, and outcomes of the patients are summarized in Table 1. The mean age was 46.1 ± 15.0 years, and sixty-three patients (68.5%) were males. A total of 79 patients (85.9%) had a witness present during cardiac arrest, and 61 patients (66.3%) received cardiopulmonary resuscitation by bystanders. In the first monitored rhythm, a shockable rhythm was identified in 66 patients (71.7%). The mean time from arrest to ROSC was 21.5 ± 13.9 minutes. Twenty-eight patients had scores ≥24 on the initial MMSE, and their median MMSE score was 25.5 (IQR, 24.0–28.0). Of the 64 cognitively impaired patients, 53 patients underwent a follow-up MMSE in the ICU.

### 3.1. MMSE Scores Immediately after Regaining Consciousness

**Figure 2:**
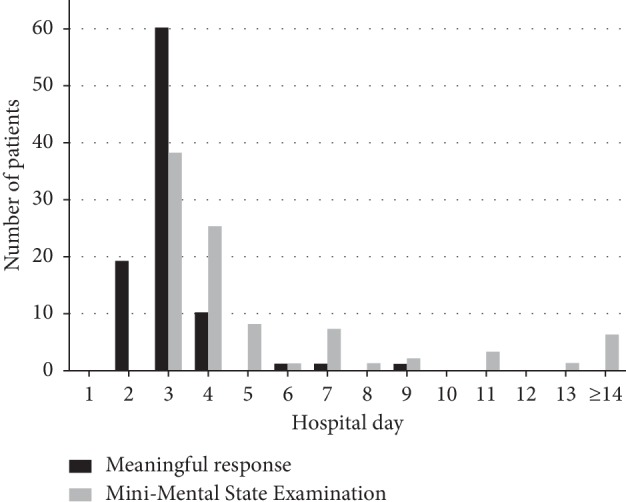
Time to meaningful response and first Mini-Mental State Examination among the participants.

Figure 2 shows the time to obey and the time to the first MMSE in all participants. A meaningful response was presented by patients at a median time of day 3 (IQR, 3.0–3.0) of the hospital stay, and the first MMSE was administered at a median time of day 4 (IQR, 3.0–5.0) of the hospital stay. The median MMSE score was 21.0 (IQR, 16.0–24.0), and cognitive impairment (MMSE < 24) was found in 69.6% (*n* = 64) of patients. The median scores in each of the 6 domains were as follows: 2.0 for orientation to time (IQR, 1.0–3.0), 5.0 for orientation to place (IQR, 3.0–5.0), 3.0 for registration (IQR, 3.0–3.0), 3.0 for attention/calculation (IQR, 1.0–4.0), 1.0 for recall (IQR, 0.0–1.0), and 8.0 for language/visual construction (IQR, 7.0–9.0). The orientation to time, attention/calculation, and recall cognitive domains were more affected than the other domains.

### 3.2. Nature of Cognitive Recovery

**Figure 3:**
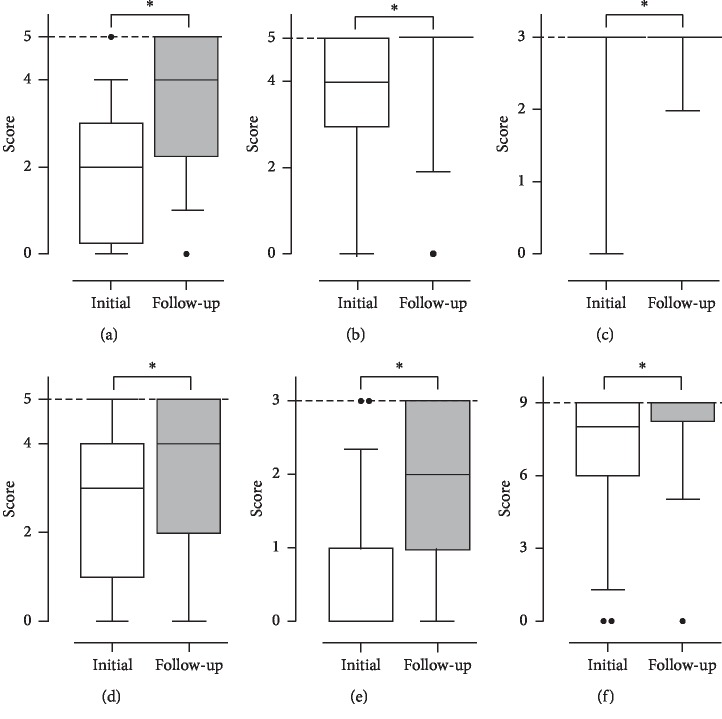
Changes in the score of each domain of Mini-Mental State Examination at 2 different time points (*n* = 53). Bars represent the median values, boxes represent the interquartile range, and whiskers extend to the 5th and 95th percentiles. Dashed lines indicate the maximal score of each domain. ^*∗*^*p* < 0.05. (a) Orientation to time. (b) Orientation to place. (c) Registration. (d) Attention/calculation. (e) Recall. (f) Language/visual construction.

A total of 53 patients completed follow-up tests, and their median scores were 20.0 (IQR, 13.5–23.0) and 25.0 (IQR, 21.5–28.0) for the first (at a median of 3 days [IQR, 3.0–4.0]) and last tests (at a median of 6 days [IQR, 5.0–8.0]), respectively. The difference between the two test scores was statistically significant (*p* < 0.001). In all 6 domains, a significant improvement in scores was observed (orientation to time: 2.0 [IQR, 0.3–3.0] to 4.0 [IQR, 2.3–5.0], *p* < 0.001; orientation to place: 4.0 [IQR, 3.0–5.0] to 5.0 [IQR, 5.0–5.0], *p* < 0.001; registration: 3.0 [IQR, 3.0–3.0] to 3.0 [IQR, 3.0–3.0], *p*=0.015; attention/calculation: 3.0 [IQR, 1.0–4.0] to 4.0 [IQR, 2.0–5.0], *p* < 0.001; recall: 0.0 [IQR, 0.0–1.0] to 2.0 [IQR, 1.0–3.0], *p* < 0.001; language/visual construction: 8.0 [IQR, 6.0–9.0] to 9.0 [IQR, 8.3–9.0], *p* < 0.001; Figure 3). More than half of the patients recovered maximal scores in the orientation to place, registration, and language/visual construction domains. In contrast, attention/calculation and recall exhibited less recovery than the other cognitive domains.

### 3.3. Factors Associated with Cognitive Impairments

When all participants were divided by their initial MMSE scores into two groups, there was only a statistically significant difference between the groups in age at the time of cardiac arrest (*p*=0.020; Table 1). We also divided the patients according to initial and last MMSE scores for patients who had a follow-up MMSE (*n* = 53) (Table 2). According to the dichotomization using the initial MMSE scores, there was no significant difference between the groups. On the other hand, for last MMSE score, the cognitively impaired group was significantly older than cognitively intact group (49.7 ± 13.4 years vs. 40.9 ± 12.6 years, *p*=0.023). The time to ROSC and the time to the last MMSE were also longer in the cognitively impaired group than cognitively intact group (*p*=0.004 and *p*=0.041, respectively). Finally, the length of hospital stay in the cognitively impaired group was also longer than that of the cognitively intact group (*p*=0.013).

To evaluate the independent predictors of cognitive impairment, we adjusted for age, time to ROSC, and LOS on the multivariate analysis (Table 3). In the analysis including all participants, age was significantly associated with cognitive impairment on the initial MMSE (OR, 1.04 [95% CI, 1.01–1.07], *p*=0.028). In contrast, in the patients who also had a follow-up test, age was not associated with an initial lower MMSE score. Factors that were correlated with a lower MMSE score on the last MMSE were older age (OR, 1.07 [95% CI, 1.01–1.14], *p*=0.016), increased time to ROSC (OR, 1.08 [95% CI, 1.02–1.15], *p*=0.012), and LOS (OR, 1.07 [95% CI, 1.00–1.14], *p*=0.044).

### 3.4. MMSE Score and Serum Biomarkers

NSE and the S100B serum levels were analysed as two groups that were divided according to each test time (Table 4). Scores from the first cognitive assessment were not associated with the biomarker levels. In contrast, the serum levels of both NSE and S100B at 24 h (*p*=0.030 and *p*=0.022, respectively) and of S100B at 48 h (*p*=0.009) were significantly higher in the cognitively impaired group than in the cognitively intact group according to the last MMSE score.

## 4. Discussion

In our study, the incidence of cognitive impairment immediately after regaining consciousness, based on an initial MMSE score < 24, was 69.6%. In addition, we revealed that recall, orientation to time, and attention/calculation were initially the more impaired cognitive domains. Although the follow-up MMSE showed that these domains recovered substantially, the recall and attention/calculation domains still were impaired even before ICU discharge. Age and time to ROSC were independent predictors of cognitive impairment on the follow-up MMSE, which was associated with high serum biomarker levels.

In our results, the early time course of cognitive dysfunction was dynamic. Although more than two-thirds of all patients had scores <24 on the initial MMSE, a significant overall improvement was observed from the initial to the final MMSE among patients who underwent a follow-up test. The change in MMSE score was a 5-point increase over 3 days (median values). Finally, more than two-thirds had scores ≥24 points. This clinical course of early-phase cognitive impairment would be important information for counselling families.

Significant differences among the initial scores of each MMSE domain were also observed. Memory function, particularly recall, was preferentially impaired. The functions of orientation to time and attention/calculation were moderately to severely impaired. Although these declines recovered substantially over a short period, the recall and attention/calculation domains were still impaired even before ICU discharge.

Traditionally, the MMSE was used as a screening instrument for cognitive impairment in ICU [5, 15, 16]. Although recently MoCA is used as representative congitive exam in cardiac arrest survivors, the MoCA took nearly twice as longer to perform than the MMSE [20], and patients who cannot satisfactorily complete the MMSE are likely to experience unnecessary stress while completing more in-depth and intense exams [12]. Therefore, Koller et al. [12] presented that MMSE testing should still be utilized as a screening tool prior to the administration of exams such as the MoCA. We also believed that it is appropriate to use the MMSE to screen cognitive function immediately after regaining consciousness and to evaluate clinical course of cognitive impairment in a short period.

Brain anoxia after cardiac arrest causes severe brain injury in survivors. To the best of our knowledge, the present study is the first clinical report to describe the quantitative association between cognitive impairment and time to ROSC in patients treated with TTM. Following a brief period of circulatory arrest, a patient may be transiently confused or may develop a severe Korsakoff-like amnesic state with profoundly impaired recall and recognition abilities but a retained short-term memory [21]. Amnesia following cardiac arrest is associated with limited lesions affecting the bilateral hippocampus with little cortical damage [22, 23]. Recovery after a prolonged arrest is associated with intellectual deficits, including disorders of attention, orientation, insight, and judgement [24]. In our results, increasing time to ROSC was correlated with cognitive impairment on the last MMSE (at a median time of hospital day 6).

Most of the previous investigations of the incidence of cognitive decline after cardiac arrest assumed that impairments were specifically related to brain anoxia. However, brain anoxia may not be the only or even the predominant cause of mild cognitive impairment that is observed during ICU care. In addition to neuronal injury by hypoxic insult, another possible cause is the use of sedatives during TTM. In the setting of hypothermia, decreased metabolic activity is also believed to delay the clearance of sedatives [18]. Interestingly, our results showed that the total administered dose of midazolam was not different between cognitively impaired and intact groups. Moreover, different serum levels of biomarkers between groups according to last cognitive function shows that neuronal injury may play a role in these cognitive impairments, which is consistent with the previous literature [25]. Grubb et al. investigated the prognostic value of serum protein S100B and NSE concentrations for predicting memory impairment using RBMT at discharge and reported that correlation coefficients for RBMT score versus serum S100B levels, especially between 24h and 48 h after ROSC was significant [25].

On the other hand, the immediate cognitive dysfunction was lesser likely to be associated with hypoxic brain injury and it suggests that there may be another explanation for the immediate cognitive decline. In not only postcardiac arrest patients but also postoperative patients, cognitive declines were commonly observed [26]. Microemboli released during the surgery have been widely assumed to be the principal cause, but few studies have shown a robust correlation between the number of emboli and cognitive outcomes [27]. Although the pathophysiology of postoperative cognitive dysfunction remains poorly understood, it is considered to be a multifactorial process by nonspecific stress [26, 27]. Like this, it could be hypothesized that nonspecific multifactorial effects of major stress, including chest compression, medications, hypothermia, and artificial ventilation, might also be contributing factors to initial cognitive decline in our cohort.

Lilja et al. recently reported that similar levels of cognitive impairment were found in cardiac arrest survivors treated with TTM and a matched control group of ST-segment-elevation myocardial infarction patients who had the same cardiovascular risk factors [9]. In studies of patients who underwent coronary artery bypass grafting (CABG) and matched controls, the intervention itself was not responsible for the cognitive impairment, and the initial decline of cognitive function reflected a more severe stage of the underlying cardiac disease necessitating CABG [27]. Our cohort was composed of relatively young patients who had relatively low frequencies of these comorbidities such as hypertension, DM, and CAD. Although no differences were observed regarding comorbidities between the two groups, we could not clarify the impact of the cardiovascular burden on cognitive impairment in this study.

Many who achieved ROSC will suffer from the postcardiac arrest syndrome, a highly inflammatory state characterized by reperfusion injury and oxidative stress, and it affects not only brain injury but also multiorgan dysfunction [28]. Moreover, TTM has an impact on all biological processes. Infection including sepsis, acute kindey injury, electrolyte abnormalities was reported as adverse events during intensive care of these patients [29]. These general conditions may affect cognitive function and consequentially MMSE score. Although our retrospective study could not analyse these conditions as confounder, we believed that the association of longer LOS with last cognitive dysfunction might be due to these conditions.

Our cognitive assessments were conducted in a short period, and there are several limitations to interpreting the results. The first limitation is that this study was a retrospective, registry-based study, which may decrease the generalizability of the results. MMSE was not performed for all consciousness survivors. In addition, a follow-up test was not performed in some patients with cognitive dysfunction. Although midazolam dosage was reduced during rewarming and stopped before reaching normal body temperature, there was no further detailed protocol. Thus, the results become difficult to interpret and generalize. Second, our results were analysed without knowledge of the baseline cognitive functions of the participants. The educational levels of patients, including the average number of years attending school, was not considered a confounding variable, and neuropsychiatric comorbidities such as previous stroke, cognitive impairment, and other psychiatric disorder, which would have impact on the MMSE more than cardiovascular comorbidities, were also not evaluated. We did not evaluate cognitive complaints via subjective questionnaires or questionnaires from the partners. A subjective sense of cognitive decline cannot be detected by standardized neuropsychological testing, and such self-reported cognitive symptoms most commonly involve memory, which was the most affected domain in our results. Third, in some delayed awakening patients, sedatives were used for critical care after TTM, but we could not adjust the analyses to control for sedative administration. Thus, the results of this study should be cautiously interpreted, and a further prospective study including various cognitive function tests is needed to increase the generalizability of our results.

## 5. Conclusion

In this study conducted in the ICU, more than two-thirds of the survivors exhibited cognitive impairment immediately after regaining consciousness, based on an MMSE score < 24. In addition, the impaired cognitive domains identified from the MMSE were recall, orientation to time, and attention/calculation. The follow-up MMSE showed that these domains recovered substantially, but some patients still had impaired cognitive function in the recall and attention/calculation domains even before ICU discharge. Older age, the time to ROSC, and LOS seemed to be associated with cognitive impairment on the follow-up MMSE.

## Figures and Tables

**Table 1:** Patient characteristics and initial MMSE scores in total participants (*n* = 92).

	MMSE ≥ 24 (*n* = 28)	MMSE < 24 (*n* = 64)	*p*
Demographics			
Male	20 (71.4)	43 (67.2)	0.687
Age, years	40.6 ± 13.0	48.5 ± 15.3	0.020
Comorbidities			
CAD	3 (10.7)	7 (10.9)	0.975
CHF	1 (3.6)	1 (1.6)	0.543
Arrhythmia	0 (0.0)	3 (4.7)	0.244
Hypertension	4 (14.3)	13 (20.3)	0.493
Diabetes mellitus	0 (0.0)	5 (7.8)	0.128
Malignancy	0 (0.0)	1 (1.6)	0.506
OHCA	24 (85.7)	56 (87.5)	0.815
Cardiac cause	25 (89.3)	56 (87.5)	0.808
Shockable rhythm	22 (78.3)	44 (68.8)	0.336
Witnessed	25 (89.3)	54 (84.4)	0.534
Bystander CPR	21 (75.0)	40 (62.5)	0.243
Time to ROSC, min	19.6 ± 11.8	22.3 ± 14.8	0.396
Midazolam dose, mg^*∗*^	271.1 (184.5–342.9)	222.8 (162.6–319.1)	0.242
Time to obey, day^*∗*^	3.0 (2.3–3.0)	3.0 (3.0–3.0)	0.637
Time to exam, day^*∗*^	4.0 (3.0–6.5)	4.0 (3.0–5.0)	0.449
Time interval from last midazolam to exam, day^*∗*^	1.0 (0.0–3.8)	1.0 (0.0–2.0)	0.751
LOS, day^*∗*^	11.5 (9.0–17.0)	14.5 (9.0–21.8)	0.377
MMSE score^*∗*^	25.5 (24.0–28.0)	18.0 (13.3–21.0)	<0.001
Orientation to time	3.5 (3.0–4.0)	1.5 (0.3–3.0)	<0.001
Orientation to place	5.0 (4.3–5.0)	4.0 (3.0–5.0)	0.006
Registration	3.0 (3.0–3.0)	3.0 (3.0–3.0)	0.011
Attention/calculation	4.0 (3.3–5.0)	1.0 (0.3–3.0)	<0.001
Recall	2.0 (1.0–3.0)	0.0 (0.0–1.0)	<0.001
Language/visual construction	9.0 (9.0–9.0)	8.0 (6.0–9.0)	<0.001

^*∗*^Median (interquartile range); MMSE, Mini-Mental State Examination; OHCA, out-of-hospital cardiac arrest; CAD, coronary artery disease; CHF, congestive heart failure; CPR, cardiopulmonary resuscitation; ROSC, return of spontaneous circulation; LOS, length of hospital stay.

**Table 2:** Patient characteristics according to first and last MMSE scores among participants with a follow-up MMSE (*n* = 53).

	Initial MMSE score			Last MMSE score		
	MMSE ≥ 24 (*n* = 12)	MMSE < 24 (*n* = 41)	*p*	MMSE ≥ 24 (*n* = 35)	MMSE < 24 (*n* = 18)	*p*
Demographics						
Male	7 (58.3)	29 (70.7)	0.418	23 (65.7)	13 (72.2)	0.631
Age, years	42.6 ± 12.2	44.3 ± 13.9	0.698	40.9 ± 12.6	49.7 ± 13.4	0.023
Comorbidities						
CAD	1 (8.3)	4 (9.8)	0.882	4 (11.4)	1 (5.6)	0.488
CHF	0 (0.0)	1 (2.4)	0.585	1 (2.9)	0 (0.0)	0.469
Arrhythmia	0 (0.0)	3 (7.3)	0.335	1 (2.9)	2 (11.1)	0.218
Hypertension	3 (25.0)	5 (12.2)	0.276	5 (14.3)	3 (16.7)	0.819
Diabetes mellitus	0 (0.0)	1 (2.4)	0.585	1 (2.9)	0 (0.0)	0.469
Malignancy	0 (0.0)	1 (2.4)	0.585	1 (2.9)	0 (0.0)	0.469
OHCA	10 (83.3)	38 (92.7)	0.330	31 (88.6)	17 (94.4)	0.488
Cardiac cause	10 (83.3)	38 (92.7)	0.330	33 (94.3)	15 (83.3)	0.196
Shockable rhythm	8 (66.7)	31 (75.6)	0.537	28 (80.0)	11 (61.1)	0.140
Witnessed	10 (83.3)	32 (78.0)	0.691	29 (82.9)	13 (72.2)	0.366
Bystander CPR	9 (75.0)	23 (56.1)	0.239	23 (65.7)	9 (50.0)	0.268
Time to ROSC, min	15.6 ± 8.3	20.6 ± 12.7	0.207	16.1 ± 11.7	25.9 ± 9.9	0.004
Midazolam dose, mg^*∗*^	236.3 (184.5–286.3)	239.7 (170.9–344.4)	0.774	242.0 (190.7–342.8)	208.6 (133.6–342.2)	0.419
Time to obey, day^*∗*^	3.0 (2.0–3.0)	3.0 (3.0–3.0)	0.151	3.0 (3.0–3.0)	3.0 (3.0–3.0)	0.335
Time to exam, day^*∗*^	3.5 (3.0–4.0)	3.0 (3.0–4.0)	0.981	5.0 (5.0–7.0)	7.0 (5.0–12.5)	0.041
Time interval from last midazolam to exam, day^*∗*^	1.0 (0.0–1.8)	1.0 (0.0–1.0)	0.901	2.0 (2.0–4.0)	5.0 (2.0–10.3)	0.011
LOS, day^*∗*^	11.5 (9.0–16.3)	15.0 (9.5–21.0)	0.139	13.0 (9.0–17.0)	16.0 (13.0–34.5)	0.013

^∗^Median (interquartile range).

**Table 3:** Independent predictors for cognitive impairment (MMSE < 24).

	All participants (*n* = 92)		Participants with follow-up MMSE (*n* = 53)			
			Initial MMSE score		Last MMSE score	
	Adjusted OR^†^ (95% CI)	*p*	Adjusted OR^†^ (95% CI)<	*p*	Adjusted OR^†^ (95% CI)	*p*
Age, years	1.04 (1.01–1.07)	0.025	1.01 (0.96–1.06)	0.750	1.07 (1.01–1.14)	0.016
Time to ROSC, min	1.01 (0.98–1.05)	0.498	1.03 (0.96–1.10)	0.433	1.08 (1.02–1.15)	0.012
LOS, day	1.02 (0.98–1.06)	0.298	1.07 (0.97–1.19)	0.181	1.07 (1.00–1.14)	0.044

^†^ORs are adjusted for age, time to return of spontaneous circulation, and time to cognitive assessment. MMSE, Mini-Mental State Examination; OR, odds ratio; CI, confidence interval; ROSC, return of spontaneous circulation.

**Table 4:** Comparison of serum biomarkers between patients with and without cognitive impairment (*n* = 53).

	First cognitive assessment			Last cognitive assessment		
	MMSE ≥ 24 (*n* = 12)	MMSE < 24 (*n* = 41)	*p*	MMSE ≥ 24 (*n* = 35)	MMSE < 24 (*n* = 18)	*p*
NSE 0 h, ng/mL (*n* = 48)	22.03 (15.30–31.52)	18.59 (15.08–22.80)	0.206	19.09 (13.84–23.05)	20.05 (16.85–26.66)	0.301
NSE 24 h, ng/mL (*n* = 45)	19.94 (15.67–24.10)	19.20 (15.16–22.27)	0.653	17.86 (14.95–21.80)	21.40 (18.02–26.19)	0.030
NSE 48 h, ng/mL (*n* = 37)	11.27 (7.66–17.48)	12.40 (8.17–17.33)	0.604	11.58 (7.98–17.44)	15.20 (7.86–17.29)	0.425
NSE 72 h, ng/mL (*n* = 23)	9.70 (8.01–19.86)	11.29 (10.51–12.90)	0.804	10.78 (8.30–13.85)	13.64 (11.09–13.65)	0.315
S100B 0 h, ng/mL (*n* = 51)	0.27 (0.18–1.62)	0.29 (0.15–0.95)	0.464	0.23 (0.17–0.67)	0.37 (0.15–1.75)	0.411
S100B 24 h, ng/mL (*n* = 51)	0.08 (0.06–0.09)	0.09 (0.06–0.11)	0.484	0.07 (0.05–0.10)	0.09 (0.07–0.13)	0.022
S100B 48 h, ng/mL (*n* = 46)	0.07 (0.06–0.10)	0.10 (0.06–0.12)	0.395	0.07 (0.06–0.11)	0.11 (0.08–0.15)	0.009
S100B 72 h, ng/mL (*n* = 25)	0.07 (0.04–0.08)	0.06 (0.04–0.09)	0.912	0.06 (0.04–0.08)	0.08 (0.06–0.11)	0.111

MMSE, Mini-Mental State Examination; NSE, neuron-specific enolase; S100B, S100 calcium-binding protein B.

## Data Availability

The data used to support the findings of this study are available from the corresponding author upon request.
